# Innovative Approaches to Clinical Data Management in Resource Limited Settings Using Open-Source Technologies

**DOI:** 10.1371/journal.pntd.0003134

**Published:** 2014-09-11

**Authors:** Raymond Omollo, Michael Ochieng, Brian Mutinda, Truphosa Omollo, Rhoda Owiti, Seth Okeyo, Monique Wasunna, Tansy Edwards

**Affiliations:** 1 Drugs for Neglected Diseases *initiative* Africa, Regional Office, Nairobi, Kenya; 2 Centre for Clinical Research, Kenya Medical Research Institute, Nairobi, Kenya; 3 MRC Tropical Epidemiology Group, London School of Hygiene &Tropical Medicine, London, United Kingdom; University of Oxford, United Kingdom

## Introduction

The primary objective of clinical data management (CDM) is to provide high-quality, reliable data for reporting randomized controlled trials (RCTs) in line with good clinical practice (GCP) requirements. For treatment trials of neglected tropical diseases (NTDs) in endemic countries, CDM systems need to be efficient and affordable. Challenges include poor infrastructure, license costs associated with GCP-compliant software, and limited human resources to provide the required expertise. We argue that high-quality CDM for NTDs can be achieved and that challenges can be overcome through the use of readily available open-access tools.

The Drugs for Neglected Diseases *initiative* (DND*i*) [1], [2] sponsors several RCTs of treatments for visceral leishmaniasis (VL) in eastern Africa, working with regional partners through the Leishmaniasis East Africa Platform (LEAP). These trials are conducted according to GCP standards to ensure internal and external validity, with CDM conducted by the authors at the Data Centre (DC) based at DND*i* Africa in Nairobi.

Choice of proprietary or open-source tools is dependent largely on budget and user experience, with tools used independently or in combination. We have been working to overcome two main challenges:

1. Telecommunication, in particular with unstable internet connectivity. An ideal tool for CDM would allow implementation with or without an internet connection (online and offline modes respectively) and be configured for use across multiple remote sites.
2. Automated query generation and resolution to provide an audit trail ensuring consistency, rigor, and limited risk of human error.

We describe our development of two new tools for CDM: an offline module for OpenClinica users and a query management system (QMS) that allows automation and standardization of query management with multiple-user access.

## Use of Open-Source Tools for CDM in Remote Settings

A popular tool for RCTs is OpenClinica [3], a GCP-compliant database package used in our DC since 2009. OpenClinica Community Edition has many advantages for CDM but was only available to use in an online mode. Without a validated offline mode of working with OpenClinica, the system's ability to support multicenter trials is limited to paper data collection and centralized data entry. Offline electronic data capture (EDC) in line with GCP would lead to substantial reductions in time between data entry and database lock [4].

**Figure 1:**
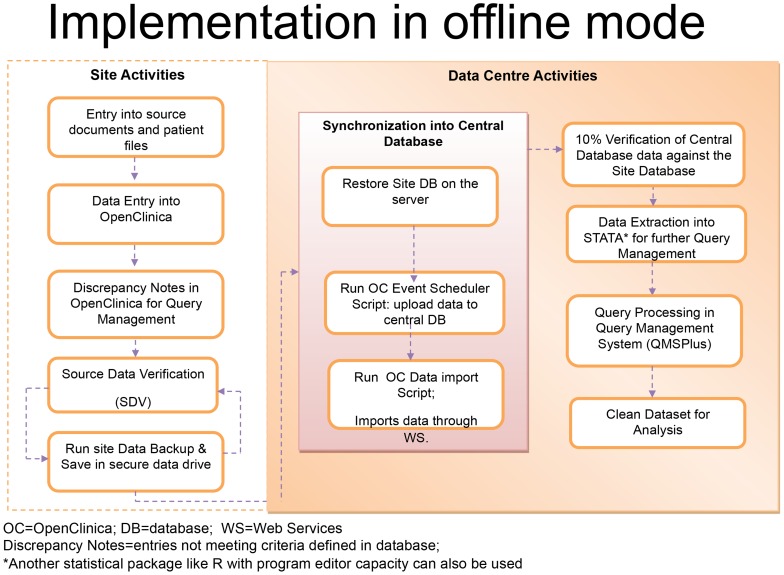
Process flow for the implementation of OpenClinica in offline mode.

We developed a validated offline module for OpenClinica to meet GCP requirements and to be made freely available to the user community (Figure 1, Table 1). Data synchronization was enabled by developing codes using python scripts (Table S1), coupled with dedicated technical support and user training. As a result, actual entry of data into the system in a remote setting is faster because the delays associated with poor connectivity are eliminated and the time taken for the data to reach the DC is shortened due to the fact that paper case report forms (CRFs) do not require physical transportation, site capacity has been strengthened, and the site staff feel engaged with the project beyond patient management through participation in CDM.

**Table 1:** Innovative tools.

OpenClinica in Offline Mode:	QMSPlus:
**Advantages**	**Advantages**
• Internet connectivity with high bandwidth not needed during data entry and only periodic connectivity needed for synchronization, so data entry process is much faster.	• More efficient because of reduction in data load on the main study database and multiple-user capacity.
• Development of an on-site data management capacity and improved motivation.	• Automated, standardized query management, improving efficiency and reducing the risk of human error.
• Potential to simplify for adaption for local and national data capture.	• Potential to adapt into open-access tool for wider use, e.g., local and national data capture with simple automated, standardized reporting output.
**Disadvantages**	**Disadvantages**
• Need for remote data support to sites.	• Set up of the trial-specific edit check programming is time consuming, which is common with any system.
• Complex synchronization process done at setup.	• Currently uses a proprietary statistical software package which could inhibit development of open-access package.

Our tool builds offline functionality into OpenClinica to be freely available to community users. This differs from the integration tool of Mi-Forms created by the Mi-Corporation [5]. Mi-Forms require installation of new external software.

## A New Approach to Query Management

**Figure 2:**
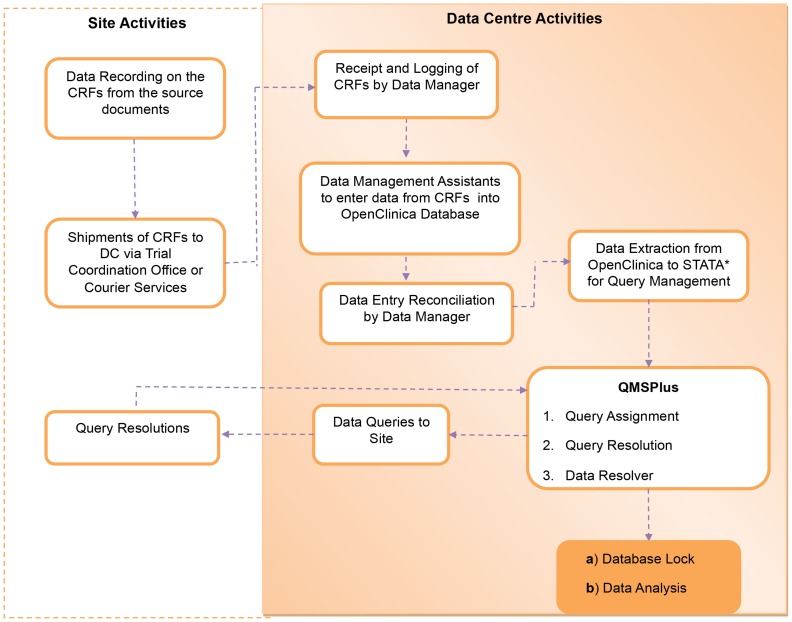
Process flow for query management within the QMSPlus. *Another statistical package like R can also be used.

Because of the large volume of data generated in VL trials (>1000 fields per patient), comprehensive cross-checking and query identification in OpenClinica would cause severe operational delays even with strong, stable internet connectivity. We used OpenClinica for entry and basic consistency checks and developed the Query Management System Plus (QMSPlus) (Figure 2 and Table 1). Following data entry validation, the system exports data into a statistical package with a program editor function that can save large amounts of code to run edit checks (for this component, we use STATA [6] based on a historical preference).There are three main modules in QMSPlus: (1) Query Assignment, which produces standardized query reports to be sent to different destinations depending on the nature of the query (site, trial, or data manager); (2) Query Resolution for data correction; and (3) Data Resolver, which produces programs to update the STATA database. These processes are repeated until a satisfactory response is achieved for all queries raised. Modules 1 and 3 are fully automated; module 2 is semiautomated in that data resolution values must be entered manually into the system on an ongoing basis as resolutions are received from the sites.

QMSPlus is a web-based Java application using PostgreSQL, allowing multiple users access to the database simultaneously. The data output from the QMSPlus (module 3) can also be exported to any other statistical analysis package with a program editor function to therefore be applicable for query management irrespective of preference of analysis software. With further development, QMSPlus could become an open-source tool if open-source statistical software such as the R package (Table 2) is used in place of STATA.

**Table 2:** Key online resources.

Resource	Location
**Data Management Tools:**	
OpenClinica: open-source database software.	www.openclinica.com
OpenClinica offline module: a user-written module to allow data entry without internet connectivity.	http://en.wikibooks.org/wiki/OpenClinica_User_Manual/OfflineDistributions
	http://blog.openclinica.com/2013/07/17/another-option-for-using-openclinica-in-disconnected-settings/
R: R is a language and environment for statistical computing and graphics and is available as free software under the terms of the Free Software Foundation's GNU General Public License in source code form.	www.r-project.org
**Regulatory:**	
US Food and Drug Administration Guidelines for Computerized Systems Used in Clinical Investigations.	www.fda.gov/OHRMS/DOCKETS/98fr/04d-0440-gdl0002.PDF
International Conference of Harmonization Good Clinical Practice (ICH GCP): GCP for Data Management.	International Conference on Harmonisation. *ICH Expert Working Group: Guideline for Good Clinical Practice, E6*, Section 5.1.3; 1996.
**Networks:**	
Society of Clinical Data Managers (SCDM).	www.scdm.org/
Global Health Trials.	http://globalhealthtrials.tghn.org
Association for Data Management in the Tropics(ADMIT).	http://admit.tghn.org

## Discussion

We have developed two innovative tools to enhance the productivity and rigor of our CDM systems: the offline mode for OpenClinica and the QMSPlus. We have intentionally utilized open-source tools to demonstrate that high-quality, GCP-compliant CDM is possible in endemic countries. We demonstrate that it is possible to overcome connectivity challenges and move beyond a spreadsheet system of queries with its associated drawbacks such as lack of authentication and audit trail, single-user use, formatting constraints, and maintenance challenges [7]. We estimate that QMSPlus has substantially reduced query management turnaround time by approximately 60%. Previously, 100 queries would take at least a day to transcribe onto the data clarification forms (DCF) before sending to the sites. Now the same volume takes just 2–3 hours to review and electronically send to the sites for resolutions. In the future, it should be possible to replace STATA in the QMSPlus program, which requires purchase of a license, with an alternative open-source package in order for QMSPlus to be made available as an open-access tool.

Since resources are needed for training, open-source software is not necessarily cost-free, but this expense is justifiable when compared to the cost of proprietary tools for noncommercial research [8]. Within the DC, the skill sets available for the implementation of these innovative approaches are primarily in data management, programming, system validation, and statistics, with experience in CDM for clinical trials. Knowledge sharing could lead to jointly created validation instruments and sharing of best practices.

Further adaptations could enable local data capture of information relating to patient case load, response to treatment and safety reporting to Ministries of Health, and for pharmacovigilance studies. Simplified systems could facilitate standardized data collection, reporting of prevalence surveys, and mass treatment coverage, ideally using mobile devices. National control programs will need to capture, collate, and report vast amounts of data on disease prevalence and treatment coverage as scale-up of mass treatment distribution for NTDs gains momentum and monitoring of progress towards elimination goals is required [9].

## Conclusion

Through the innovative use of open-source tools, knowledge sharing, and strengthening of research networks, high-quality data management is possible for all those working towards improved control of NTDs [10] while adhering to the principles of GCP.

## Supporting Information

Table S1 Implementing OpenClinica in offline mode.
(DOCX)
