# Supplementary material for: Innovative Approaches to Clinical Data Management in Resource Limited Settings Using Open-Source Technologies
Source: PLoS Negl Trop Dis. 2014 Sep 11;8(9):e3134. doi: 10.1371/journal.pntd.0003134 (PMC4161312; doi:10.1371/journal.pntd.0003134)
Supplement: Table S1 — Implementing OpenClinica in offline mode. (DOCX) [file pntd.0003134.s001.docx]

**Supplementary Table 1: Implementing OpenClinica in Offline mode**

| **Key Processes**   - Study database setup on the main study server: Create the production study database and define study sites and site users. This becomes the main or central study database against which routine synchronization of site/offline instances will be done. The central database dump is then used to replicate the database on all site computers. - Proceed to collect data at the sites: Once the database is installed on the site computers, the site users can proceed with data entry normally. No internet connection is needed since OpenClinica is locally installed on the site computers. Data are then periodically extracted from the study computers in CDISC ODM 1.3 Xml format and sent to the DC where a central database is located for import. A database dump is also generated by the site and sent to the DC. - Synchronization at the DC: When the Data Center receives both the extracted data and the database dump, the database dump is plugged into Postgres database on the same server as the Central study database. This means that a database with “site name” as part of the filename is created on the same server as central database and the site database dump restored on it. A python script (see <http://www.python.org>) referred to as OC Event Scheduler is executed. The script synchronizes data for distinct study subjects between the site and central databases accordingly. The Script also synchronizes subject’s events which will then make it possible to import the extracted site data into the central database using the OpenClinica data import functionality. - Alternatively, OC Event Scheduler and Data Import scripts can be configured to execute directly from the site computer hence synchronizing with the central database through OpenClinica web services, without having to send extract data from the site computer the site database dump to the data center. - The data import can either be done manually or a system task can be scheduled to automate the entire process.   **Requirements:**  1. Besides the standard OpenClinica software stack, Python Software (from <http://www.python.org/download/releases/2.7.2/>) must be installed in order to execute the OC Event Scheduler script.  2. Psycopg2 (PostgreSQL database adapter for the Python programming language) is available for download from <http://initd.org/pub/software/psycopg2> and also <http://www.stickpeople.com/projects/python/win-psycopg/> for Windows. |
| --- |
